# Early Parental Loss and Self-Rated Health of Older Women and Men: A Population-Based, Multi-Country Study

**DOI:** 10.1371/journal.pone.0120762

**Published:** 2015-04-01

**Authors:** Susan P. Phillips, Lisa Carver

**Affiliations:** 1 Department of Family Medicine, Queen’s University, Kingston, Ontario, Canada, and Umeå Centre for Gender Studies, Umeå University, Umeå, Sweden; 2 Department of Sociology, Queen’s University, Kingston, Ontario, Canada; University of Westminster, UNITED KINGDOM

## Abstract

**Objective:**

Death of a parent in childhood can diminish both the nurturing that promotes healthy development, and household income. We consider, for the first time, whether this adverse childhood experience is associated with self-rated health decades later, among seniors and whether this lifelong effect is different for women and men.

**Methods:**

The International Mobility in Aging (IMIAS) study is a prospective cohort with survey information and biophysical measures and markers from 2000 community-dwelling 65–74 year olds in Canada, Colombia, Brazil and Albania. We assessed the independent impact of death of a parent, early hunger, and witnessing violence, while controlling for current income sufficiency and other early adversities on self-rated health using baseline (2012) IMIAS data. Regressions grouping and then separating women and men were compared.

**Results:**

Approximately 17% of the 1991 participants had experienced early parental loss. Overall 56% rated their health as good however parental loss predicted poorer adult health, as did early hunger but not witnessing violence. Disaggregated analyses revealed that the health consequences of parental loss were significant only among men (p = 0.000 versus p = 0.210 for women) whereas early hunger predicted poor self-rated health for both (p = 0.000).

**Conclusion:**

Parental loss should be considered as a potent adverse childhood experience with life-long consequences for health. The gender difference in its effect, speaks to unidentified and modifiable traits that appear to be more common among women and that may build resilience to long-term harms of early parental death.

## Introduction

Early social and economic experiences can and do embed themselves in the body, with effects that ‘bubble up’ years later when opportunities to ameliorate any adversity are long past[1]. The harms to health of these adverse experiences in childhood (ACEs) appear to be cumulative and enduring [2–4]. Some refer to this writing of social and environmental events on individual biology as embodiment while others describe the phenomenon in terms of environmental epigenetic switches that ultimately activate and/or suppress genes [5,6]. The empirical evidence from which both these explanatory theories arise is increasing and consistent. Those whose early life was marked by violence, neglect, or hunger, to name a few ACEs, suffer disproportionately from self-reported and objectively measured mental and physical illness decades later. On the other hand, individual traits and assets that collectively augment the ability to bounce back and thrive in the face of a significant threat, that is, traits that foster resilience can modify the long-term impact of ACEs [6]. In particular, a nurturing adult (usually but not necessarily a parent) who helps a child build strengths rather than capitulate in the face of life’s challenges can mitigate the harms of early adversity [7].

Death of a parent removes or minimizes a source of protective nurturing from a child’s life as well as potentially precipitating socioeconomic deprivation if the deceased parent were the family’s primary income earner [8,9]. Since nurturing by a caring adult is central to successful psychological and biochemical navigation of threats presented by adverse childhood experiences early parental death could pose a particular long-term risk to health [10]. Never the less, the loss of a parent during childhood has not previously been considered as a predictor of older adult self-rated health (SRH) [11]. Early parental loss has been shown to increase short-term vulnerability among children, and to predict poorer subsequent mental health and increased mortality in early adulthood [12]. However, among 18–65 year olds in the Netherlands death of a parent was the only early adversity of nine that were studied that did not decrease health-related quality of life [13]. In this paper we ask whether death of one or both parents prior to age 15 has an independent impact on self-rated health decades later among those living in four developed and developing countries.

## Methods

### Context

The International Mobility in Aging Study (IMIAS) is a prospective cohort study with the primary objective of examining how events across the life-course affect the health, and particularly, the mobility, of people age 65–74. By recruiting equal numbers of men and women across four developing and developed countries the differential impact of gender and social circumstances on health can be studied. Each setting has a relatively homogeneous older population, however there is a high level of socioeconomic and cultural variability across sites. Tirana, a city of approximately 700,000, is the capital of Albania, a post-communist country and one of the poorest in Europe. Natal in coastal northeastern Brazil, is a provincial capital with a population of approximately 800,000. The research setting in Colombia was Manizales (population 400,000), a mountainous city in a coffee-growing region. Kingston, Canada is a small, primarily English-speaking city of 110,000 with a stable, non-industrial economy while St. Hyacinthe, also in Canada, is a relatively traditional Francophone community of about 50,000 and with an agricultural economic base. Canada is a developed and relatively high-income country while the other three are considered middle-income settings.

### Population and Sampling

This analysis uses baseline cross-sectional data from IMIAS, collected in 2012. Community-dwelling men and women, age 65–74 at the time of the initial survey (2012) were recruited via primary care providers’ rosters until 200 women and 200 men were enrolled at each site [Kingston (Ontario, Canada), St. Hyacinthe (Quebec, Canada), Natal (Brazil), Manizales (Colombia) and Tirana (Albania)].

At Canadian sites ethics guidelines precluded approaching perspective participants directly. Instead recruitment letters were sent via a sample of urban family physicians to all patients who met age criteria and lived independently within the city. Letters invited interested recipients to phone a research coordinator. This process yielded a response of approximately 30%. Of those who initiated participation in this manner, approximately 95% were enrolled. At the other sites ethics approval was received to approach randomly selected clinic patients directly, yielding a participation rate of greater than 90% (Colombia) and almost 100% (Brazil and Albania). All respondents were screened for cognitive ability and excluded if they had 4 or more errors on the Leganes Cognitive Test [14].

Participants were interviewed and asked all questions in the appropriate language in their homes, unless they requested a different setting. Questionnaires were available in English, French, Portuguese, Spanish and Albanian. Interviewers across all sites received standardized training by the study coordinator and/or principal investigator. Scales used within the questionnaire were all previously validated. Translations were checked for accuracy via ‘back’ translation and scale translation was validated in pilot studies in Brazil, Colombia, and Canada.[15] All responses were entered immediately by the interviewer into a computer-based program and subsequently reviewed by one of the investigators to check for possible entry errors.

### Data Collected

Embedded in a detailed survey of demographics, social circumstances, health status, subjective and objective measures of mobility, physical health, mental function, cognitive status, etc were the following questions of relevance to this paper:

Did your mother, father or both parents die during the first 15 years of your life?

During the first 15 years of your life, would you say that there was a time in which you did not eat enough and that you were hungry?

Did you ever witness physical violence between those close to you (such as between your parents, or your parents and siblings)?

To what extent does your income allow you to meet your needs?

Self-rated health was measured as excellent, very good, good, fair or poor and then dichotomized with the first three categories being collapsed as “good” and the latter two as “poor”. The positioning of the question re SRH within the overall survey was the same for all participants.

### Statistical Analyses

Using SPSS (v21) we examined Spearman correlations then built a logistic regression model using dichotomized indicators with SRH as the outcome and first with the 3 adversity measures alone then subsequently controlling for current income sufficiency. The model was then adjusted for other measures of early adversity (divorce of parents, childhood health, physical abuse in childhood). The same regression analyses were then repeated disaggregating women and men. As the number of participants with missing data was less than 1% they were excluded from relevant analyses. P-values < 0.05 were considered statistically significant.

### Ethics/consent

The IMIAS project received ethics approval from the Research Centres of the University of Montreal (CR-CHUM), the Albanian Institute of Public Health, and the Research Ethics Boards of the Federal University of Rio Grande do Norte (Brazil), Queen’s University (Kingston) and University of Caldas (Columbia). All study participants provided written informed consent and were told they could withdraw at any time. In Brazil and Colombia, for participants who could not read and write, the consent form was read to them, oral consent was obtained, and the participant placed a sign on the form to acknowledge agreement.

## Results

**Fig 1:**
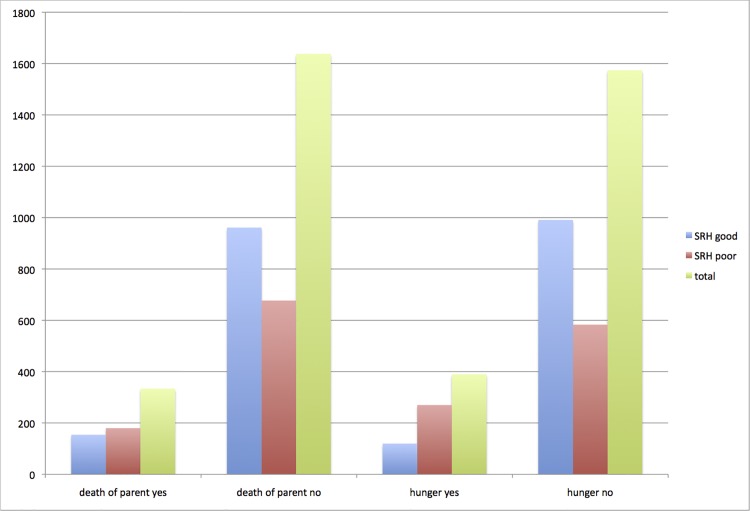
Numbers reporting good/poor self-rated health with or without either early parental death or early hunger. The y axis represents number of participants while bars are coded as follows: blue = good SRH, red = poor SRH, green = total.

A total of 1991 individuals completed the baseline IMIAS survey in 2012. Self-rated health was reported as good by 56.4% (n = 1123) overall, with more men (60%) than women (53%) rating their health as good. Childhood adversity was not unknown among the IMIAS cohort, whether their experience was loss of a parent (17%, n = 334), hunger while growing up (20%, n = 390), or witnessing violence (13%, n = 258). Early parental death was more prevalent in the middle-income countries studied with proportions reporting this loss as follows: St. Hyacinthe (10%), Kingston (11%), Albania (18%), Colombia (24%) and Brazil (25%). The relationships between loss of a parent or early hunger, and current self-rated health were then assessed (Fig. 1). Poor health was reported by 54% of those whose parent(s) died when the participant was young compared with only 41% of the group with no such loss. Similarly, poor SRH was more common among those who either experienced childhood hunger (69% versus 37% of those without early hunger) or witnessed violence (53% versus 42% of those not witnessing violence). A majority reported current income sufficiency (64.1%) compared with 717 (31.9%) whose income was not adequate for their needs.

As all measures of early and current circumstances were categorical rather than continuous, Spearman correlations were calculated but interpreted with the caution necessary for such analyses Table 1. Although each correlation coefficient is weak the large sample size results in statistical significance of the relationships between death of a parent and hunger but not parental death and witnessing violence. Current income insufficiency appears correlated with each ACE considered, although, again, the coefficients are small.

**Table 1:** Correlations among dichotomized independent variables.

	Witness violence	Death of parents	Hunger	Income sufficiency
**Witness violence**	1.00	.02 (n = 1954)	.17^a^ (n = 1948)	−.03^b^ (n = 1967)
**Death of parent(s)**	—	1.00	.11^a^ (n = 1949)	−.09^a^ (n = 1974)
**Childhood hunger**	—	—	1.00	−.19^a^ (n = 1966)
**Income sufficiency**	—	—	—	1.00

For all variables yes = 1, no = 0

^a^Correlation is significant at the .01 level (2-tailed).

^b^Correlation is significant at the .05 level (2-tailed).

Partial correlations were assessed controlling for income sufficiency to determine whether the impact of each ACE on the outcome of interest was direct, or mediated by current socioeconomic status. The correlations remained essentially unchanged and, again, weak despite being statistically significant.

Logistic regression demonstrated that when considered alone, each independent variable strongly predicted SRH: death of a parent—SRH (p = 0.000); hunger—SRH (p = 0.000); witnessing violence—SRH (p = 0.001); income sufficiency—SRH (p = 0.000). We next included the three ACEs and examined independent associations with self-rated health without controlling for current income sufficiency. The adverse experience of childhood of primary interest in this study, that is, death of a parent, remained a significant predictor of SRH (p = 0.001). The impact of early hunger appeared to be of even greater significance (p = 0.000). Witnessing violence in childhood was, however, no longer significantly associated with SRH (p = 0.111). To further assess whether the apparent impact of early hunger and death of a parent on subsequent health was direct or mediated via current socioeconomic status we then controlled for income sufficiency with results shown in Table 2. Death of a parent and hunger remained significant independent predictors of later SRH. The relationship between current income status and self reported health was also statistically significant (p = 0.000) suggesting that current income and health are linked via a pathway that does not involve the three ACEs. Adjusting for other measures of possible early adversity including childhood health, divorce of parents, and physical abuse did not alter the strong association between death of a parent and self-rated health decades later.

**Table 2:** Significance of specific adverse childhood experiences and self-rated health in older age controlling for current income sufficiency.

	Sig.	Odds ratio	95%CI lower	95%CI upper
**Income sufficency**	.000	.292	.238	.357
**Death of parent**	.015	1.381	1.064	1.791
**Hunger**	.000	3.088	2.392	3.986
**Witness violence**	.085	1.296	.965	1.741

**Fig 2:**
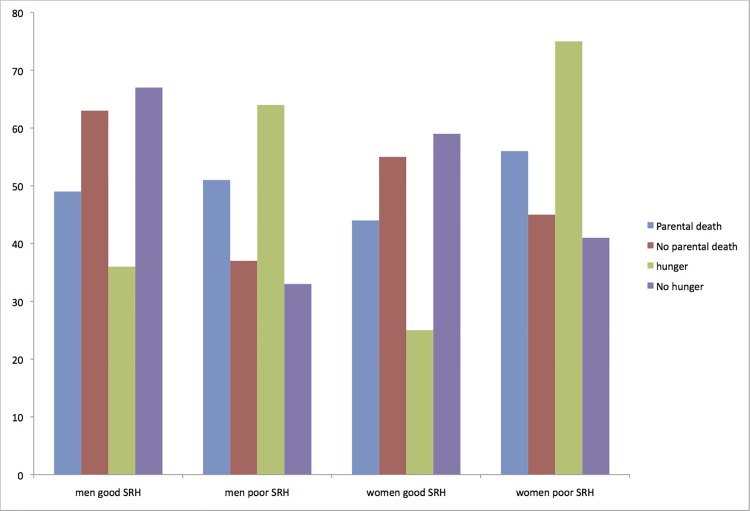
Percentages of men and women experiencing early parental death or early hunger with current good/ poor self-rated health. The y axis represents percentages while bars are coded as follows: blue = parental death, red = no parental death, green = hunger and purple = no hunger.

To assess whether early adversity might have a gender-specific impact on later SRH we repeated analyses separating the sample into the subsets, ‘men’ and ‘women’ and calculated gender-specific proportions for parental loss or early hunger, and good/poor self-rated health (Fig. 2). Poor self-rated health was reported by 51% of men and 56% of women whose parent(s) died when the participant was less than 15 years old, compared with 37% (men) and 45% (women) in the subgroup with no early parental loss. For those who experienced childhood hunger, adult SRH was poor among 64% of men and 75% of women. Those who did not suffer from hunger in childhood reported current SRH as poor less frequently (men 33%, women 41%).

Table 3 illustrates the results of logistic regression using the overall model (ie independent variables are the 3 measures of early adversity, controlling for current income sufficiency, dependent variable = SRH) but disaggregating the data by gender. Among men both early hunger and parental loss have a significant impact on current self-rated health, independent of the harm associated with present-day income insufficiency. Women appear at least as susceptible to the long-term harm of early hunger, but in contrast to men, the association between parental loss and current self-rated health is not statistically significant.

**Table 3:** Gender-disaggregated regression analyses controlling for income sufficiency.

		Odds Ratio	Sig.	95% C.I.	
				Lower	Upper
**Men**	**Death of parent**	1.490	.042	1.014	2.187
	**Early hunger**	2.965	.000	2.087	4.211
	**Witnessing violence**	1.519	.072	.964	2.394
	**Income sufficiency**	.317	.000	.236	.427
**Women**	**Death of parent**	1.255	.210	.879	1.792
	**Early hunger**	3.380	.000	2.312	4.941
	**Witnessing violence**	1.096	.645	.742	1.619
	**Income sufficiency**	.275	.000	.208	.365

## Discussion

Among the participants in the IMIAS study, 17% had lost one or both parents by age 15. This proportion varied across sites for reasons that cannot be ascertained from the data available. Our research identifies that this early parental loss is associated with poorer self-rated health in old age. The association is statistically significant for men but not women, is not mediated by current income sufficiency and remains after adjusting for a number of other measures of childhood adversity.

### Possible limitations

SRH is a subjective measure but one that has repeatedly been shown to correlate well with actual health [16]. It is a measure that is robust across culture, place and age [17,18]. In general women tend to report poorer health than do men however these gender differences reflect disparities in ability and function [19].

Although recall bias is a potential risk in asking about events that occurred 5 or 6 decades earlier, our main indicator, loss of a parent, is unlikely to be forgotten, whereas early hunger or witnessing violence, and current income sufficiency are more subjective.

With any quantitative research the question of underpowered or overpowered arises. Did we find significant differences only because of a large sample size or miss meaningful differences because the study was underpowered? The lack of previous research on parental loss and SRH in older populations precluded power and sample size planning.

IMIAS is a descriptive study therefore causal inferences cannot be drawn. It would be erroneous to generalize from our findings, which should be viewed as preliminary. Evidence from more settings and across a wider age range is needed before any suggestion of causality would be reasonable. To determine whether IMIAS participants are representative of their communities we compared their socioeconomic and education levels with available data for each city. The Kingston cohort, and particularly women within it, had more education than did non-participants of the same age range in their community. Latin American participants were representative of the SES and educational status of their cities. Never the less, because the populations sampled may not be representative, we have avoided analyzing site-specific data or claiming that findings have external validity.

Interviews in homes meant that in settings outside Canada there were often family members present. As a result, respondents may have been guarded about describing, for example, current family violence. However, the responses about childhood experiences analyzed in this paper are unlikely to have been answered differently had there been privacy.

The multi-country nature of the study ensures socioeconomic and cultural diversity among participants. However, this variability can only be accurately captured in analyses within measures of specific characteristics. Site specific reporting has been avoided for several reasons. We cannot assume that samples are representative of each community’s population nor can we make inferences about possible community characteristics that give rise to or mediate self-rated health. Furthermore, this study lacks the power to assess associations between parental loss and SRH by site.

### Strengths and Interpretations

In bivariate analyses each of the three ACEs predicted poorer self-rated adult health, as did insufficient current income. Death of a parent remained a significant predictor after controlling for other ACEs and for current income sufficiency. Early death of a parent may rob a child of nurturing, stability, a sense of normalcy, and the income that parent would have provided. Although each of these deprivations, alone, can have a significant and long-lasting impact on mental and physical health, to date, parental loss has not specifically been studied as a predictor of adult self-rated health. Our study indicates that death of a parent in childhood may create a real and ongoing wound. Its negative health impact is only minimally ameliorated by income sufficiency in later life. This would suggest that parental loss may be an independent predictor of SRH decades later and that its effect is not mediated via current socioeconomic status.

The impacts of parental loss and early hunger on later health are both statistically significant but different. Those with adequate intake in childhood are more likely to rate their current health as good (63% vs 57% of the whole cohort) while early hunger predicts poorer SRH than that described by the cohort, overall (69% vs 43%). On the other hand, growing up with 2 parents alive does not particularly improve adult SRH (59% of good SRH among those with two parents alive vs 57% overall) while loss of a parent is clearly associated with poorer health (54% poor SRH among those with parental loss vs 43% of total cohort). Data, alone cannot explain why having two parents in childhood does not confer measureable benefits while parental loss translates into poorer lifetime self-rated health. It may be that in the face of this adversity some children bounce back and thrive, that is, they develop a resilience that their two parent counterparts lack, and that this resilience mitigates the health harms of parental loss. The health detriment of early hunger may be more direct than that of a parent’s death and not corrected by developing resilience. This could explain why the absence of hunger bestows a health benefit not seen for the absence of parental death.

The impact of ACEs on adult health was markedly different for women and men. A disproportion of women report poor SRH overall, in keeping with findings across other studies and settings [19,20]. However the relative impact of early parental loss on later SRH is much greater for men than women, whereas both suffer significant long-term consequences of early hunger regardless of current socioeconomic status. The gender difference in long-term health is in keeping with findings of increased cortisol responses among men relative to women aged < 55 following early parental loss [21]. We hypothesize that the harmful impact of this particular adversity is modified by unidentified individual and/or social circumstances that, themselves, vary by gender. It is not early parental death, alone, that writes the story of harm on the body foecades to come but the interplay of this loss and individual and socially mediated strengths, constraints, expectations and opportunities of those who suffered the loss. Some unidentified circumstance(s) operating in conjunction with but not limited to death of a parent, appear to decrease the harmful impact on women’s health and/or magnify it among men. Our data cannot further define this gender difference. It may be that young men are more susceptible to the loss of nurturing that parental death brings and/or that women are more able to translate loss into inner strength and resilience [21]. As Krieger states in describing eco-social and embodiment theory: “how people resist injustice and its health-harming effects, individually and collectively, and the resilience that enables them to do so also must be examined” [5].

Existing research on gender differences in ACEs is limited but suggests that women tend to be more prone to later harm from early adversity [22]. The somewhat greater long-term effect of early hunger on health among the women in our cohort is in keeping with this. However, loss of a parent in childhood is not among the ACEs previously studied. Our results provide preliminary evidence that the harm to later health arising from death of a parent may be mediated by a different set of gendered characteristics than those described for other ACEs.

This study is the first to report that parental loss should be considered as a childhood adversity with lifelong consequences for self-rated health and, further, that these consequences are and can be ameliorated or modified [23]. By identifying what decreases the impact of loss on subsequent health for women relative to men it may be possible to develop individual or community-based interventions that augment those beneficial characteristics in general. The observed gender differences on health long after parental loss suggest that childhood adversities do not inevitably harm but that other aspects of one’s life, environment and circumstances modify and mitigate this association. What those aspects are remains unidentified. We refer to them collectively as resilience and in subsequent IMIAS data collection will include measures of current resilience. However such measures give only an immediate snapshot of characteristics when resilience measured at a time more proximal to the loss itself might be of greater explanatory value. The traits that build resilience vary over time and most meaningful would be measures of these done much closer to the time of parental loss. Such measures cannot be collected retrospectively. This makes the argument for including measures of resilience in prospective, longitudinal population-based health surveys to actually identify factors linked to resilience and whether their presence moderates the ills associated with adversity.

In summary, we have documented the significant and long-lasting harm to self-rated health associated with loss of a parent in childhood. The health impact of this loss is greater among men than women. This gender difference suggests that the detrimental effect of death of a parent in childhood may be modified by individual and/or social traits.
