# Correction: Early Parental Loss and Self-Rated Health of Older Women and Men: A Population-Based, Multi-Country Study

**DOI:** 10.1371/journal.pone.0128168

**Published:** 2015-05-21

**Authors:** Susan P. Phillips, Lisa Carver

## Notice of Republication

This article was republished on May 5^th^, 2015, to remove confidential or copyrighted information. Please download this article again to view the correct version. The Data Availability statement has been corrected to read: Due to ethical restrictions imposed by the Queens University Ethics Review Board, IMIAS data are available to external interested researchers upon request. Requests may be sent to the Corresponding Author.
